# Disruption of large-scale electrophysiological networks in stroke patients with visuospatial neglect

**DOI:** 10.1162/netn_a_00210

**Published:** 2022-02-01

**Authors:** Tomas Ros, Abele Michela, Anaïs Mayer, Anne Bellmann, Philippe Vuadens, Victorine Zermatten, Arnaud Saj, Patrik Vuilleumier

**Affiliations:** Department of Neuroscience, University of Geneva, Geneva, Switzerland; CIBM Center for Biomedical Imaging, Geneva University Hospitals, Geneva, Switzerland; Romand Clinic of Readaptation, SUVA, Sion, Switzerland; Rehabilitation Clinic Valais de Coeur, Sion, Switzerland; Department of Neurology, Geneva University Hospital, Geneva, Switzerland

**Keywords:** Stroke, EEG, Hemineglect, Functional network connectivity, Alpha, sLORETA

## Abstract

Stroke frequently produces attentional dysfunctions including symptoms of hemispatial neglect, which is characterized by a breakdown of awareness for the contralesional hemispace. Recent studies with functional MRI (fMRI) suggest that hemineglect patients display abnormal *intra*- and *interhemispheric* functional connectivity. However, since stroke is a vascular disorder and fMRI signals remain sensitive to nonneuronal (i.e., vascular) coupling, more direct demonstrations of neural network dysfunction in hemispatial neglect are warranted. Here, we utilize electroencephalogram (EEG) source imaging to uncover differences in resting-state network organization between patients with right hemispheric stroke (*N* = 15) and age-matched, healthy controls (*N* = 27), and determine the relationship between hemineglect symptoms and brain network organization. We estimated *intra*- and *interregional* differences in cortical communication by calculating the spectral power and amplitude envelope correlations of narrow-band EEG oscillations. We first observed focal frequency-slowing within the right posterior cortical regions, reflected in relative delta/theta power increases and alpha/beta/gamma decreases. Secondly, nodes within the right temporal and parietal cortex consistently displayed anomalous intra- and interhemispheric coupling, stronger in delta and gamma bands, and weaker in theta, alpha, and beta bands. Finally, a significant association was observed between the severity of left-hemispace search deficits (e.g., cancellation test omissions) and reduced functional connectivity within the alpha and beta bands. In sum, our novel results validate the hypothesis of large-scale cortical network disruption following stroke and reinforce the proposal that abnormal brain oscillations may be intimately involved in the pathophysiology of visuospatial neglect.

## Author Summary

Stroke patients often exhibit a disabling deficit of visual awareness in the hemifield opposite to their brain lesion, known as hemineglect. Recent studies with functional MRI (fMRI) suggest that hemineglect patients display abnormal functional coupling (i.e., connectivity) within and between brain hemispheres. However, since stroke is a vascular disorder and fMRI measures nonneuronal (i.e., vascular) coupling, we here provide direct evidence of neural network dysfunction in hemineglect by using electroencephalogram (EEG) source imaging, which measures the electrical fluctuations of large neuronal populations. Overall, we observed a breakdown of interhemispheric network connectivity within alpha/beta rhythms, which specifically correlated with the degree of patients’ hemispatial errors. The high temporal resolution and frequency content of EEG signals could lead to more sensitive markers and targeted rehabilitation approaches of hemineglect.

## INTRODUCTION

Apart from motor or sensory impairments, the sequelae of ischemic stroke may cause a significant impact on attentional function. This is most apparent when stroke damage leads to symptoms of hemispatial neglect (Domínguez-Borràs, Armony, Maravita, Driver, & Vuilleumier, 2013; P. O. Vuilleumier & Rafal, 2000), which is characterized by an inability to attend to and process information from the left (or more rarely right) side of space (i.e., contralateral to the lesion site). Hence, right-hemisphere stroke patients exhibit an impairment in detecting visual (or auditory) stimuli in their left hemifield (and vice versa for patients with left hemisphere stroke). Despite the fact that this is a major source of disability in patients’ daily life, current treatments for hemineglect remain minimally effective. Moreover, untreated hemineglect leads to poorer recovery prognosis and reduced benefits from rehabilitation therapies for other neurological deficits.

Therefore, a deeper understanding of this clinical phenomenon is required for several interdependent reasons: (a) to better identify the neurobiological targets for rehabilitation; (b) to determine the underlying pathophysiological anomalies, in particular whether local and/or distributed brain dysfunction is crucially implicated; and (c) to elucidate the neural mechanisms that give rise to perceptual consciousness.

To address these issues, in line with recent work, here we investigate how brain activity dynamics is altered following focal hemispheric stroke, at both local and global levels, and what is the functional impact of such changes on attentional performance in patients. To this aim, we use electroencephalogram (EEG) recording and adopt a network framework by reducing the whole brain to a manageable number of regions of interest (ROIs; nodes), whose functional activity is considered to covary with each other through pairs-wise connections (edges). Utilizing this approach, emerging work has suggested that hemineglect might be associated with significant disruptions of brain functional connectivity. Specifically, several functional neuroimaging studies reported that the visuospatial deficits in hemineglect are accompanied with abnormal interhemispheric and/or intrahemispheric connectivity (Baldassarre et al., 2014; Fellrath, Mottaz, Schnider, Guggisberg, & Ptak, 2016; Guggisberg et al., 2014; Sasaki et al., 2013; Yordanova et al., 2016). Most recently, using functional MRI (fMRI), Ramsey and colleagues showed that recovery from hemineglect is linked to the return of previously depressed interhemispheric connectivity between nodes of sensorimotor and attention networks (Ramsey et al., 2016). However, since stroke is a vascular disorder and fMRI signals remain sensitive to nonneuronal (i.e., vascular) coupling, stronger validation of neural network dysfunction in hemineglect is warranted. Physiologically speaking, fMRI measures local changes in brain metabolism, which generally echo (but lag by a few seconds) electrophysiological activation/deactivation patterns of neuronal populations (Bentley, Li, Snyder, Raichle, & Snyder, 2016; Hermes, Nguyen, & Winawer, 2017; Hutchison, Hashemi, Gati, Menon, & Everling, 2015). Interestingly, studies comparing noninvasive electrophysiological methods such as magnetoencephalography/electroencephalography (M/EEG) with fMRI found a tight spatiotemporal correspondence between the amplitude envelopes of neural oscillations and neurovascular fMRI signals (Brookes et al., 2011; de Pasquale et al., 2010; Mantini, Perrucci, Del Gratta, Romani, & Corbetta, 2007), although there are several instances of dissociations between fMRI and neuronal signals (Schölvinck, Maier, Ye, Duyn, & Leopold, 2010). Furthermore, abundant research in both human and nonhuman primates has linked specific oscillatory activities with attentional functions in the normal brain, particularly implicating modulations of the alpha range in relation to selective visuospatial orienting (Marshall, Bergmann, & Jensen, 2015b), but also the theta and beta ranges in relation to task-dependent interactions across networks (Bauer et al., 2012; Fiebelkorn, Pinsk, & Kastner, 2018), as well as the gamma band in relation to local intra-areal processing of sensory information (Szczepanski et al., 2014). However, how such coordinated oscillatory activities are disrupted by stroke and affected by clinical neglect deficits still remain unresolved.

Given the linkage between M/EEG and fMRI signals and their relation to attentional processes, the goal of our current study was to investigate the neural substrates of hemineglect using source space EEG, which reflects genuine neuroelectric activity uncontaminated by vascular dynamics. Specifically, we performed (a) functional whole-brain connectivity comparisons between stroke patients and healthy controls, and (b) regression analyses to identify changes connectivity patterns more selectively associated with visuospatial deficits (i.e., perceptual omissions during search tasks) in the left and right hemifields, respectively.

## RESULTS

### Group Functional Connectivity in Patients Compared to Healthy Elderly Controls

**Figure 1.**
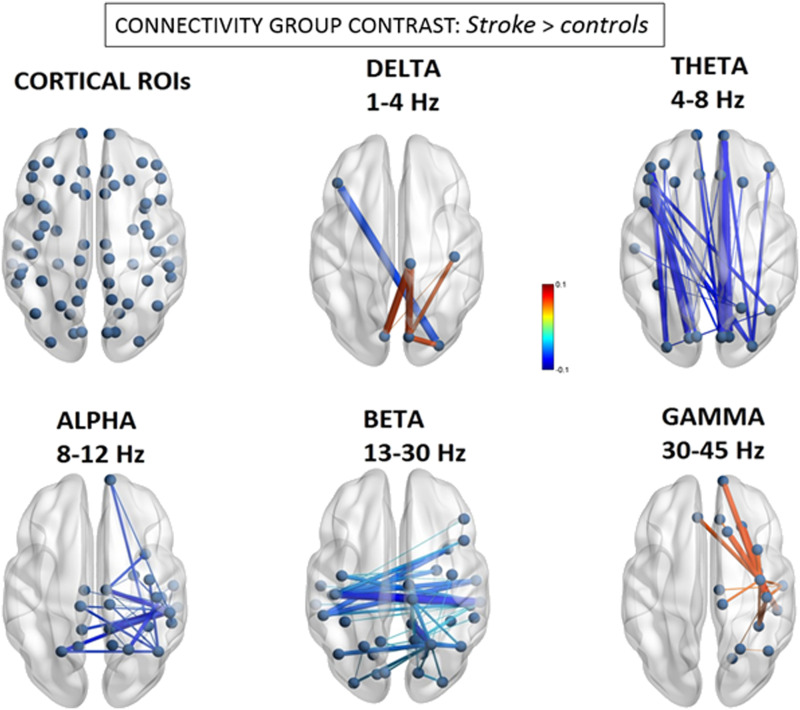
Group differences in EEG source functional connectivity. Significant differences of the magnitude of amplitude envelope correlations between stroke patients and controls. Line thickness represents larger absolute connectivity difference (Pearson’s correlation coefficient *r*). Red color indicates greater connectivity for patients versus controls, while blue color indicates greater connectivity for controls versus patients (only significant connectivity components are displayed, *p* < 0.05 network-based statistic corrected).

We defined the EEG source space functional network connectivity (FNC) graphs for both the stroke patients (*n* = 15) and the age-matched healthy controls (*n* = 27), and then contrasted the graphs from each group in order to test for changes in connectivity patterns across the different frequency bands. As illustrated in Figure 1, this revealed three main patterns in the patients relative to controls: (a) the delta band showed a *mixed pattern* of both increased and decreased FNC; (b) the theta-, alpha-, and beta- bands all displayed *decreased* FNC, but with distinctive anatomical distributions; and (c) the gamma band demonstrated selective and localized *increases in* FNC.

More specifically, using a network-based statistic (NBS) correction threshold of *p* < 0.05, we found the delta band demonstrated significant but limited changes with mainly intrahemispheric hyperconnectivity, predominating between the right paracentral lobule (BA4) and occipital areas (BA18) (*d* = 0.063, *t* = 3.28; *d* = −0.063, *t* = 3.58, for sources in right and left cuneus, respectively), but also a more limited interhemispheric hypoconnectivity between the right lateral occipital cortex (BA18) and left inferior prefrontal cortex/pars triangularis (BA45) nodes (*d* = −0.064, *t* = −3.36).

For the theta band, we observed mainly long-range, intrahemispheric hypoconnectivity changes along the anterior-posterior axis that, remarkably, were present on both sides. These decreases were the largest between the left pericalcarine (BA17) and left inferior prefrontal gyrus/pars orbitalis (BA47) nodes (*d* = −0.079, *t* = −4.51), as well as between the right pericalcarine (BA17) and right frontal pole (BA11) nodes (*d* = −0.081, *t* = −4.15).

In sharp contrast, the beta band showed an opposite pattern of hypoconnectivity, mainly affecting interhemispheric connections, particularly those centered on posterior right-hemisphere (RH) areas. These interhemispheric changes affected connectivity between homologous areas of the superior parietal and temporal cortices. The greatest reductions in connectivity were found for the right hemisphere, between the right superior parietal lobule (BA7) and left posterior cingulate gyrus (*d* = −0.051, *t* = −5.59), as well as between the right middle temporal (BA20) and right precuneus (BA3) nodes (*d* = –0.041, *t* = −4.93).

Alpha-band hypoconnectivity was characterized by a more focal cluster of reduced connections emerging from the posterior right hemisphere. The most salient interhemispheric disconnectivities were observed between right posterior temporal (BA20) and left superior parietal (BA7) nodes (*d* = −0.130, *t* = −4.65), as well as between right inferior parietal (BA39) and left superior parietal (BA7) nodes (*d* = −0.126, *t* = −5.32).

Finally, the gamma band displayed a local hyperconnectivity pattern predominating mainly within the anterior right hemisphere. These difference were maximal between the right precentral (BA6) and right superior frontal (BA32) gyri (*d* = 0.065, *t* = 2.50), between the right precentral (BA6) and right inferior temporal (BA20) gyri (*d* = 0.064, *t* = 2.30), as well as between the right precentral (BA6) and right frontal pole (BA10) (*d* = 0.062, *t* = 2.35).

### Relationships Between Network Connectivity and Visuospatial Bias in Hemineglect Patients

Although group-level analyses indicated anomalous FNC between stroke patients and control subjects, they do not disambiguate which network connections, if any, may be related to the emergence of visuospatial deficits. Many of our patients presented signs of hemispatial neglect, as commonly observed in right brain–damaged patients (Verdon et al., 2010), but to a varying degree. The FNC changes described above could more generally reflect the effect of posterior stroke damage. Hence, in order to disentangle neurobehaviorally specific from nonspecific connections in stroke-associated hemineglect, we performed regression analyses directly testing for any relation between individual patients’ FNC matrices and the severity of neglect symptoms, as indexed by the number of left/right omission errors in the visual cancellation test. These errors are considered as one of the most reliable markers of neglect (Klinke, Hjaltason, & Tryggvadóttir, 2018).

Using a NBS correction threshold of *p* < 0.05, we found significant brain–behavior relationships that distinctively affected the alpha, beta, and gamma bands. Changes in the delta and theta bands did not predict the severity of visuospatial neglect.

**Figure 2.**
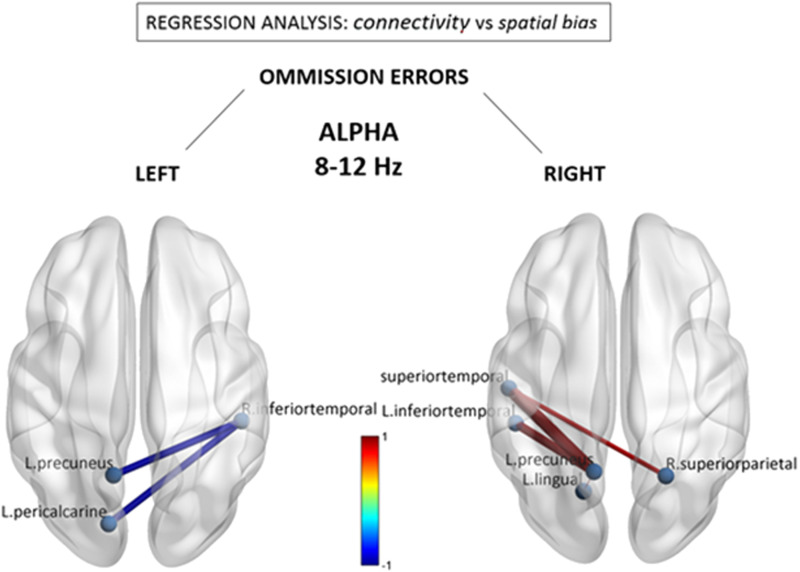
Alpha (8–12 Hz) connectivity as a function of neglect symptoms. Depicted connections correspond to changes in individual FNC components correlating with the number of omissions in the left and right hemifields, respectively, during the cancellation task. Red/blue values indicate statistically significant beta coefficients (*p* < 0.05 network-based statistic corrected). Further conjunction analysis (see text) revealed that reduced connections associated with left omissions (in left panel) were those showing the most significant overlap with general stroke-related decreases at the group level.

As seen in Figure 2, the alpha band demonstrated a remarkable contrast between omissions in left and right space, implicating near mirror image interhemispheric connections. Consistent with the group-level hypoconnectivity described above, the number of leftward misses was associated with selective *decreases* in connectivity between extrastriate visual regions in the right inferior temporal gyrus (BA20) with the left precuneus (BA3) (beta = −0.99) and left pericalcarine region (BA17) (beta = −0.98). Conversely, the number of misses within the right hemifield (typically reflecting more extensive spatial neglect) was significantly predicted by *increases* in alpha-band connectivity within a homologous set of visual and parietal nodes, which included the left inferior temporal cortex (BA20) and the left precuneus (BA3). Importantly, a separate regression with the *total number of left and right misses* (see Supporting Information Figure S2) did not reveal any shared connections with either left or right omissions, but limited reductions in connectivity between left medial parietal and right inferior frontal areas, reinforcing the idea that the changes in alpha band may be specifically related to spatially directed attentional processing.

**Figure 3.**
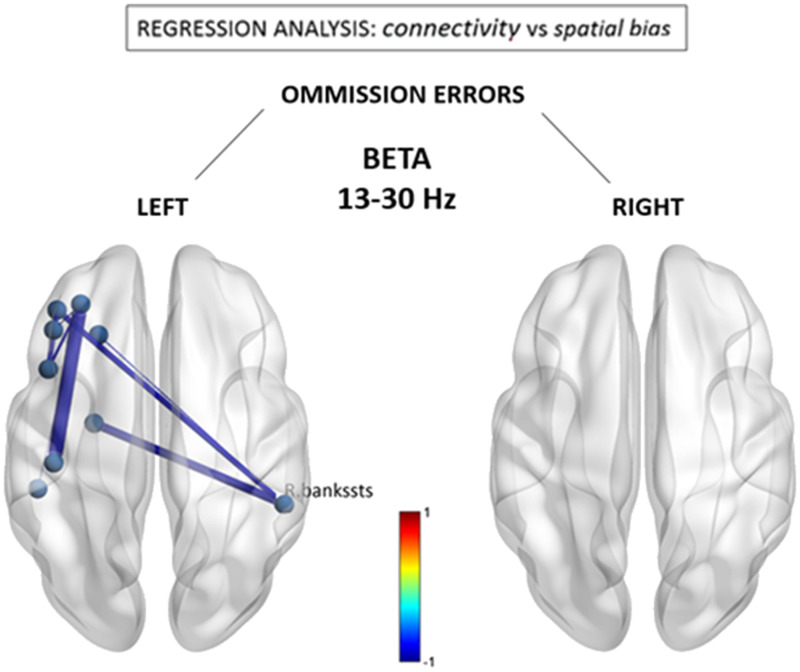
Beta (13–30 Hz) connectivity as a function of neglect symptoms. Depicted connections correspond to changes in individual FNC components correlating with the number of omissions in the left and right hemifields, respectively, during the cancellation task. Red/blue values indicate statistically significant beta coefficients (*p* < 0.05 network-based statistic corrected). R. bankssts = right bank of the superior temporal sulcus.

Figure 3 shows results from similar regression analyses for the beta band, demonstrating an association of leftward omissions with a different pattern of reduced functional connections between the right posterior-superior temporal region (BA22) and anterior brain regions in the left middle frontal gyrus (BA6) (beta = −0.67) and left inferior frontal gyrus/pars orbitalis (BA47) (beta = −0.70). Reductions were also seen within the anterior left hemisphere. No significant associations were observed for the number of rightward errors, nor were there any systematic functional connectivity changes that regressed with the total number of left and right omissions (see Supporting Information Figure S3).

**Figure 4.**
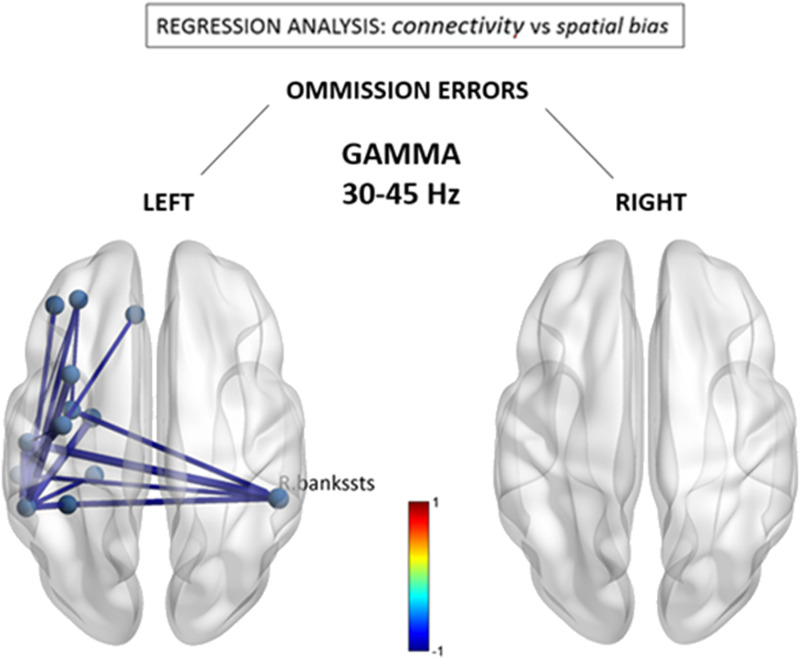
Gamma (30–40 Hz) connectivity as a function of neglect symptoms. Depicted connections correspond to changes in individual FNC components correlating with the number of omissions in the left and right hemifields, respectively, during the cancellation task. Red/blue values indicate statistically significant beta coefficients (*p* < 0.05 network-based statistic corrected). R.bankssts = right bank of the superior temporal sulcus.

Lastly, as can be seen in Figure 4, the relationship between leftward omissions and FNC within the gamma band revealed a node also implicated within the beta band, namely, the right posterior-superior temporal region (BA22) that acted as a hub for reduced connections with mirror areas in left temporal regions. Also similar to the beta band, additional reductions were seen within the anterior left hemisphere. However, surprisingly, these negative changes contrasted with the overall increases in connectivity observed for the gamma band at the group level (see Figure 1). There was no correlation between connectivity patterns in gamma band and omissions in right space.

### Conjunction Analysis Between Group Functional Connectivity Abnormalities and Correlates of Visuospatial Errors

Finally, we asked whether the abnormal functional components seen as a direct marker of stroke at the group level are consistent with those that interindividually predict stronger leftward visuospatial deficits. In other words, is there evidence for a distinctive disruption of FNC components associated with clinical symptoms of hemineglect? This question was addressed by using a standard conjunction analysis, with the aim to identify the overlapping or “in common” components that were significant in the group-wise difference test *and* in the behavioral regression test. Accordingly, a correct test for a logical AND requires that all the comparisons in the conjunction are individually significant at *p* < 0.05 (Nichols et al., 2005). Hence we performed a conjunction of tests (i.e., global null hypothesis) to identify the overlap—if any—between only the statistically significant (*p* < 0.05 corrected) group-level FNC difference maps (i.e., Figure 1) and the counterpart visuospatial regression maps (i.e., Figures 2 and 3), across the statistically significant alpha, beta, and gamma bands.

Interestingly, among all abnormal connections, we identified a single overlap with a network component that includes a node in the *right inferior temporal gyrus* (BA20, MNI coordinates X = 49, Y = −31, Z = −23) *within the alpha band*. This network component was interhemispherically disconnected with the left precuneus (BA3) and left pericalcarine region (BA17), identical to the connections illustrated in the left panel of Figure 2. This suggests that among connections globally disrupted by right hemispheric stroke, these may be the most reliable marker of left hemineglect symptoms.

### Group Spectral Power in Patients Compared to Healthy Elderly Controls

#### Absolute power.

In addition to interregional FNC measures, regional source spectral power (i.e., current source density) differences were estimated between stroke patients and controls using independent *t* tests across the five EEG bands (false discovery rate [FDR] corrected). Absolute power was consistently *more elevated* in the right (damaged) hemisphere of patients relative to controls for all EEG bands (delta, theta, beta, gamma) *except for alpha*, where no significant differences were detected (*p* < 0.05 corrected). As seen in Supporting Information Figure S4, absolute delta power was stronger within posterior parietal areas (*t* = 3.1, *p* < 0.05 corrected), theta power within widespread regions with a maximum over supramarginal regions (*t* = 3.8, *p* < 0.05 corrected), while beta was higher mostly over frontal (*t* = 2.7, *p* < 0.05 corrected), and gamma mostly over precentral motor regions (*t* = 3.8, *p* < 0.05 corrected). Supporting Information Figure S6 illustrates the absolute power spectral density of hemineglect and control groups at the sensor level (using a current source density montage at electrode P4, over the right parietal cortex). Interestingly, no significant associations were detected between absolute EEG power changes and visuospatial performance (*p* < 0.05 corrected).

#### Relative power.

We also tested for differences in relative (%) power, often used to normalize spectra under a constant value of broadband (1–40 Hz) power, and reflecting the degree of spectral slowing (i.e., greater relative power in lower frequencies) or spectral acceleration (i.e., greater relative power in higher frequencies). As evidenced by Supporting Information Figure S5, neglect was associated with relative spectral slowing of right posterior cortical regions, with relative delta power being more elevated in patients mainly within the inferior parietal/posterior temporal junction (*t* = 3.6, *p* < 0.05 corrected) while relative theta power was higher within the superior parietal cortex (*t* = 3.4, *p* < 0.05 corrected). Conversely, relative alpha power was maximally reduced within the inferior parietal/posterior temporal junction (*t* = −3.6, *p* < 0.05 corrected), in conjunction with relative beta (*t* = −4.4, *p* < 0.05 corrected) and relative gamma (*t* = −3.2, *p* < 0.05 FDR corrected) reductions within the superior parietal lobe. Supporting Information Figure S7 illustrates the relative power spectral density of hemineglect and control groups at the sensor level (using a current source density montage at electrode P4, over the right parietal cortex). No significant associations were detected between relative EEG power changes and visuospatial performance (*p* < 0.05 corrected).

### Relating Connectivity and Oscillatory Power Changes With Lesion Topology

Given that changes in functional connectivity (FC) and source power were topographically specific, a reasonable question to ask is whether connectivity differences could be explained firstly by changes in signal power? According to this hypothesis, reduced connectivity would indirectly reflect a degraded signal-to-noise ratio caused by a loss of source EEG power. The latter could result in particular from neuronal loss at the site of the stroke lesion. However, additional analyses allowed us to rule out this hypothesis, given the rather limited overlap between anatomical regions with maximal lesion extent and those with strongest disruptions of connectivity. As shown in Supporting Information Figure S8, the location of largest lesion overlap occurred in fronto-insular cortex (which was common to no more than 10 patients), encompassing regions such as BA44, BA45, and BA6. This co-localized with 5 RH nodes/parcels of the Desikan–Killiani atlas used for FNC calculations: *entorhinal*, *insula*, *temporal pole*, *superior temporal*, and *precentral* regions (see Supporting Information Figure S4 for their exact locations in MNI space). Importantly, removal of these particular nodes (or their connections) from our analysis did not alter our principal findings, which highlight alpha/beta disconnectivity with more posterior temporo-parietal foci (see Supporting Information Figure S4 for anatomical verification).

On the other hand, although regions with significant *relative* power differences (indicative of relative frequency shifts in EEG power) did partly overlap with temporo-parietal nodes presenting abnormal FC power in alpha/beta bands (see Supporting Information Figure S1), this was clearly not the case for *absolute* source power (see Supporting Information Figure S2), which provides a more direct measure of electrical signal strength. Notably, each narrow-band EEG frequency range (from delta to gamma) exhibited a unique topographical fingerprint (e.g., see Figure 2), arguing against a simple generative mechanism of reduced EEG signal-to-noise ratio due to neuronal loss that would presumably lead to a broadband attenuation of electrical activity across the full EEG frequency spectrum.

Instead, and given the partial FC overlap with nodal reductions in relative alpha/beta power, we suggest that this pair of EEG rhythms may act as carrier waves for attentional information (Wróbel, 2000) compared to other frequency channels (e.g., delta, theta, gamma), and that it is their *relative* degradation compared to other frequencies that may be an index of impaired network communication (Akam & Kullmann, 2012).

## DISCUSSION

Overall, our EEG results converge with previous M/EEG studies reporting electrocortical abnormalities in stroke patients with hemineglect (Colson, Demeurisse, Hublet, & Slachmuylder, 2001; Demeurisse, Hublet, & Paternot, 1998; Ros et al., 2017; Yordanova et al., 2016) and add novel neurophysiological evidence to support recent findings of neurometabolic (i.e., fMRI) alterations in functional brain networks associated with specific cognitive deficits after stroke (Siegel et al., 2016). Remarkably, we found widespread changes of FNC extending far beyond focal areas of structural brain lesions in RH, including connections with the opposite/intact left hemisphere (LH), with very distinctive patterns across different frequency bands. Moreover, a conjunction analysis that sought to overlap (a) group-level FC differences between patients and controls, together with (b) connections predicting the magnitude of leftward visuospatial inattention in a regression analysis, revealed a circumscribed reduction of interhemispheric FC between the posterior right temporal lobe and left precuneal/pericalcarine regions. This is consistent with and supportive of anatomical lesion studies that have long implicated right posterior temporal and inferior parietal areas as a critical site associated with visuospatial attention deficits (Chelazzi, Duncan, Miller, & Desimone, 1998; Heilman & Van Den Abell, 1980; Hillis et al., 2005; Karnath, Ferber, & Himmelbach, 2001; Mort et al., 2003; Ptak & Valenza, 2005; Vallar & Perani, 1986), and with the notion of an impaired interhemispheric balance in the pathological spatial biases associated with left hemineglect (Corbetta & Shulman, 2011; Kinsbourne, 1977; Mesulam, 1981). Importantly, the posterior temporal cortex has previously been associated with mechanisms of visuospatial awareness in both human and animal studies and found to be among the most frequent sites of brain damage in hemineglect (Karnath, Ferber, & Himmelbach, 2001). Finally, a separate regression with the *total number* of (left and right) *errors* did not reveal any overlap with connections specifically regressing with either left or right omissions, reinforcing the idea that the changes in alpha-band connectivity may be specifically related to spatially directed attentional processing.

Taken together, our results therefore reinforce the theory that network disturbances characterized by a weakened functional communication between key cortical regions is a fundamental mechanism underlying spatial neglect (Doricchi, Thiebaut de Schotten, Tomaiuolo, & Bartolomeo, 2008; Driver & Vuilleumier, 2001; P. Vuilleumier et al., 2001, 2008), including both interhemispheric and intrahemispheric disconnectivity.

### The Multiplex FNC Signature of Hemineglect

Compared to age-matched healthy subjects, our source space analyses of amplitude envelope correlations indicated that patients with RH stroke displayed a multiplex reorganization of neural connectivity that spans the full frequency range captured by EEG, involving delta, theta, alpha, beta, and gamma rhythms, and that covers both hemispheres in a frequency-specific manner. Specifically, delta FC was mainly elevated within posterior RH regions, while it was reduced between the posterior RH and anterior LH. These changes are consistent with nonspecific effects of brain lesions on focal delta activity (van Dellen et al., 2013) and they did not correlate with neglect symptoms. On the other hand, theta connectivity mainly exhibited a bilateral reduction of long-range intrahemispheric connections, partially consistent with recent work reporting reductions in fronto-parietal theta and beta coherence in other EEG studies of stroke patients with hemineglect (Fellrath et al., 2016; Yordanova et al., 2017). However, we found that the theta connectivity difference did not correlate directly with neglect severity, suggesting that previously reported changes in this band may reflect task-specific abnormalities during goal directed attention or nonspecific effects of stroke on executive aspects of attention unrelated to spatial neglect (see Fellrath et al., 2016).

In contrast, we found that alpha and beta rhythms both demonstrated important reductions in intra- and interhemispheric connectivity of posterior temporal and parietal cortices, that is, regions traditionally found to be implicated in hemineglect (Thimm, Fink, & Sturm, 2008; Umarova et al. 2011; Verdon et al., 2010). Furthermore, a subset of these connections was significantly associated with neglect symptoms, in particular those linking right temporo-parietal areas with left posterior parietal regions in the alpha band and with left prefrontal regions in the beta band (see Figures 2 and 3). Remarkably, changes in alpha-band connections significantly correlated with the severity of visuospatial biases and implicated symmetrical regions over the extrastriate visual cortex (pericalcarine and lingual), where hypoconnectivity with the LH predicted more left visual field omissions, while the converse (hypoconnectivity with the right hemisphere) was observed for right visual field omissions. This topographically specific disconnection pattern is compatible with the role of alpha rhythms in contralateral visuospatial attention (Ikkai, Dandekar, & Curtis, 2016; Lobier, Palva, & Palva, 2017; Marshall, Bergmann, & Jensen, 2015a; Okazaki et al., 2015; van Schouwenburg, Zanto, & Gazzaley, 2017), as well as previous studies on hemineglect utilizing functional (Ramsey et al., 2016; Sasaki et al., 2013) and structural (Karnath, Rennig, Johannsen, & Rorden, 2011; Vaessen et al., 2016) neuroimaging to delineate the functional neuroanatomy of visuospatial deficits. In particular, it is thought that lateralized modulation of alpha activity across hemispheres might gate sensory information flow from early visual areas to the ventral temporo-occipital stream, under top-down influences from the dorsal attention network (Geng & Mangun, 2009; Liu et al., 2014; van Diepen, Miller, Mazaheri, & Geng, 2016). Moreover, it is interesting to note that while the alpha band was implicated in the disconnectivity pattern of the posterior right temporal lobe with sensory visual areas, the beta band was distinctively involved in disconnectivity with left prefrontal areas, which presumably subserve more executive processes of attention (Antzoulatos & Miller, 2016).

Importantly, a selective depression of interhemispheric connectivity has in itself been shown to be a pathological hallmark of hemineglect in both fMRI and EEG studies (Ramsey et al., 2016; Sasaki et al., 2013), including for the alpha (Sasaki et al., 2013) and beta bands (Guggisberg et al., 2014). Furthermore, increased alpha and/or beta FC has been reported to be a predictive marker of clinical status after stroke or traumatic brain injury in patients with motor (Dubovik et al., 2012; Guggisberg et al., 2014; Kawano et al., 2017) as well as language (Castellanos et al., 2010; Guggisberg et al., 2014; Nicolo et al., 2015) impairments.

Finally, we also found connectivity changes in the gamma range, including increases within right frontal areas at the whole group level, as well as decreases between right temporo-parietal and LH regions that correlated with neglect symptoms and partly overlapped with beta changes. These divergent effects make it difficult to associate them with a clear functional role in spatial deficits after stroke. Moreover, gamma-range neuronal activity is usually associated with local interactions between nearby cortical populations rather than with long-distance interactions at network level (Vinck, Womelsdorf, Buffalo, Desimone, & Fries, 2013). Given theoretical proposals linking gamma activity to conscious perceptual processes (Melloni et al., 2007), one tentative hypothesis might be that a reduction of synchronous activity in gamma band between right posterior brain areas and left fronto-temporal networks would reflect the loss of access of spatial representations held in the right hemisphere (Karnath et al., 2001) to LH processes mediating conscious awareness and verbal report, in line with work in split-brain patients demonstrating an intimate link between conscious behavior and language abilities of the LH (Volz & Gazzaniga, 2017). Furthermore, through phase–amplitude coupling, gamma-band activity has recently been demonstrated to be modulated by both alpha and theta rhythms (observed to be disrupted above) in the service of spatial attention (Fiebelkorn et al., 2018; Jensen, Gips, Bergmann, & Bonnefond, 2014). Hence, it is possible that hemineglect constitutes a clinical syndrome resulting from a concerted disruption of a family of nested rhythms (Bonnefond, Kastner, & Jensen, 2017), rather than a single one. Within this theoretical framework, communication between two regions can be established by phase synchronization of oscillations at lower frequencies (i.e., theta, alpha, beta, <25 Hz), which serve as temporal reference frame for information carried by high-frequency activity (i.e., gamma, >40 Hz) (Szczepanski et al., 2014). However, this interpretation still remains speculative and the role of phase–amplitude coupling in neglect, if any, remains unsettled and undoubtedly needs further investigation to be clarified. Finally, given the lack of significant overlap between rightward omissions (which are specific markers of hemineglect) and group-level connectivity differences in the beta and gamma bands, it is equally possible that such reorganization may be the result of naturalistic recovery of cortical function that has occurred since the time of the stroke.

### Limitations

The first important limitation of our study is that it was not preregistered and that it lacked a patient control group *with* stroke but *without* presentation of hemineglect symptoms. This would have had the benefit of additionally controlling for a general and deleterious effect of stroke *itself* on brain activity. We acknowledge this as an important shortcoming in the strict context of our primary analysis of group-wise differences. However, beyond this comparison in the first step of our analysis, the current study was able to leverage hemineglect-specific behavior with the second, most important step of analysis (i.e., regression), as well as with its functional intersection with the group-wise stroke-related difference (i.e., conjunction). This double-step approach allowed us to exploit the full sample of patients and circumvent difficulties associated with a dichotomous diagnosis of neglect (Checketts et al., 2020). Hence, despite the lack of separate patient subgroups, we are confident that the network component(s) that ultimately survived this sequential statistical selection are not only robust but also clinically relevant. However, in light of the limited number of participants in our study, this does not exclude a potential region-of-interest bias due to the pooling of different anatomical lesion locations within this sample of stroke patients.

Secondly, as already noted above, the connectivity values may have been contaminated by degraded signal-to-noise ratio and global losses in source EEG power due to neuronal tissue damage. However, as shown by our supplementary analyses, we consider this interpretation unlikely for several reasons, most notably because connectivity changes included both decreases and increases, they did not correspond to the local peaks of *absolute* source power that constitutes a more direct measure of electrical signal strength, and distinct patterns were observed for each EEG frequency sub-band. Taken together, this argues against a single, global mechanism of reduced EEG signal-to-noise ratio due to neuronal loss. Instead, and given the partial FC overlap with nodal reductions in relative alpha/beta power, we suggest that this pair of EEG rhythms may act as major carrier waves for attentional information (Wróbel, 2000), distinct from other frequency channels (e.g., delta, theta, gamma), and hence it is their *relative* degradation compared to other frequencies that may constitute a functional index of impaired network communication (Akam & Kullmann, 2012).

Other potential limitations are reflective of the imaging modality we used, that is, EEG. Although EEG provides a direct measure of neural activity, it is most sensitive to sources within the cortical mantle. Hence, our analyses were restricted to cortical network dynamics and did not allow for reliable assessment of subcortical structures that may play an important role in the control of attention (Gitelman et al., 1999; P. Vuilleumier, 2013). Another weakness involves the relatively low number of EEG channels that constitute the 10–20 montage, raising potential concerns of excessive source localization error and/or spread, which would lead to spurious activity mixing between cortical ROIs. Here, given the clinical nature of our study, we found that the 10–20 montage met an important trade-off between EEG setup time and patient fatigue during daily rehabilitation sessions. Secondly, our methodology allows for replication and application in standard clinical settings where high-density EEG is not routinely available. In this context, a number of studies have shown that low-density montages may be used to reconstruct a considerably greater number of dipolar sources than there are sensors (i.e., electrodes) (for a review see Grech et al., 2008). In particular, a source localization simulation study with low-resolution electromagnetic tomography analysis (sLORETA) using 1,000 cortical sources (i.e., patches) of ∼1 cm^2^ (Song et al., 2015) and an electrode montage based on the 10–20 system indicated a mean localization accuracy of ∼0.5 cm and source spread of ∼0.7 cm, which remains within the lowest inter-ROI distances of the Desikan–Killiany atlas (∼1.5 cm) used in the present study. Importantly, as the Desikan–Killiany atlas contains ROIs of variable patch sizes, empirical work has not revealed a systematic bias between patch size and sLORETA reconstruction accuracy (Cosandier-Rimélé, Ramantani, Zentner, Schulze-Bonhage, & Dümpelmann, 2017). Lastly, in line with published work leveraging a similar low-density EEG montage in clinical conditions (Tokariev et al., 2018), our analysis pipeline included EEG signal orthogonalization prior to FC estimation, which has been shown to minimize any instantaneous (i.e., at zero-phase lag) activity that is spuriously shared between ROIs and that may have arisen from (low-resolution) source blurring (Brookes et al., 2011). Nevertheless, as source localization errors cannot entirely be excluded, caution must be exercised regarding the precise anatomical locations of cortical network nodes, given the use of a generic head template and low-density montage in this study.

### Conclusion

In a nutshell, our results show that the hemineglect syndrome following RH stroke is associated with a widely distributed but anatomically specific disruption of cortical network connectivity that involves temporo-occipital regions of the right hemisphere and their functional interactions both within and between the two hemispheres. In particular, losses in the connectivity of the posterior RH with left parieto-occipital cortex in alpha channels and with left prefrontal cortex in beta channels appear to be critically involved in impaired control of visuospatial attention toward the contralesional/left hemispace. Better mapping and understanding of these neurophysiological markers of hemineglect is an important step toward designing novel tools to assess poststroke deficits in patients and more effective rehabilitation approaches. For example, by directly training alpha oscillations by using neurofeedback (Mottaz et al., 2018; Ros et al., 2017) or transcranial alternating stimulation (also known as tACS) (van Schouwenburg, Sörensen, de Klerk, Reteig, & Slagter, 2018).

## MATERIALS AND METHODS

### Study Participants

Stroke patients participated after giving their written informed consent. The study was approved by Geneva State Ethics Committee and accorded with the Helsinki Declaration. Patients were admitted after a first RH stroke and consecutively recruited from a primary clinical center. We excluded patients with bilateral lesions, previous neurological or psychiatric disorders, impairment in primary visual perception (except partial visual field defect), psychiatric disorders, severe motor difficulties in the right upper limb, pusher syndrome (i.e., contralateral trunk deviation with active resistance to any attempt of external correction), or current psychotropic treatment. In total, we recruited 15 right-hemisphere-lesioned patients (mean age: 59.4; *SD*: 11.3; 2 women, 13 men) who fulfilled these criteria and were prospectively admitted to two primary clinical centers at the Clinique de Réadaptation of SUVA in Sion (https://www.crr-suva.ch) and the Foundation Valais de Coeur of Sion and Sierre (https://www.valaisdecoeur.ch).

Spatial neglect was assessed using a standard clinical battery similar to other research in our group (Saj, Cojan, Vocat, Luauté, & Vuilleumier, 2013; Vaessen, Saj, Lovblad, Gschwind, & Vuilleumier, 2016). Details on clinical tests are provided below. Time since stroke onset was 7.5 months on average (*SD* 5.3 months). Hemianopia or quadranopia was present in 4 out of 15 patients (26%).

All patients underwent structural MRI scans to delineate the location and extent of brain damage. Individual stroke lesions were manually delineated and the group average lesion map was created using the MRICron toolbox (https://people.cas.sc.edu/rorden/mricron). The maximal lesion overlap affected posterior parts of the lateral prefrontal cortex, the anterior and middle temporal lobe, as we all as the deep paraventricular white matter in the right hemisphere (see Supporting Information Figure S1).

As we sought to explore electrophysiological differences between stroke patients and the healthy population, we also collected data from a control group of 27 matched healthy adults (mean age: 56; *SD*: 7; males: 23, females: 4), randomly sampled from the Human Brain Institute (HBI) normative database (https://www.hbimed.com/) (Grin-Yatsenko, Baas, Ponomarev, & Kropotov, 2009). Importantly, the healthy subject in the HBI data were collected using the same EEG amplifier (Mitsar-201) and EEG montage (10–20 international montage) as the recordings in stroke patients.

### Clinical Battery for Visuospatial Neglect

A series of standard paper-and-pencil tasks were presented to each patient at their first visit, in order to determine the presence and severity of baseline visuospatial biases in attention. Neglect severity was measured with the Schenkenberg line bisection task (Schenkenberg, Bradford, & Ajax, 1980a) (18 horizontal lines, 10–20 cm) and a variant of the bell cancellation test (Gauthier, Dehaut, & Joanette, 1989) (35 animal targets among distractor objects) (Gassama, Deplancke, Saj, Honoré, & Rousseaux, 2011). In the latter animal cancellation test, the search sheet was divided into seven virtual columns, each containing five targets. In total, there were 15 targets in the left side, 15 in the right side, and 5 in the central column. Clinical neglect was defined as abnormal performance in the line bisection test (cutoff: rightward deviation > 11%) (Schenkenberg, Bradford, & Ajax, 1980b) and target cancellation test (cutoff: left-right omissions ≥ 4 out of 15 omissions) (Gauthier et al., 1989). However, some patients showed deficits in one of these tests only, and all those selected for our sample (*n* = 15) demonstrated some signs of hemineglect according to at least one of these tests (86% of patients had cancellation deficits, 79% had line bisection deficits), as commonly observed after RH damage (Halligan et al., 1991; Verdon et al., 2010). However, neglect severity varied substantially between patients (leftward omission error range: 13%–100%; mean: 77%; *SD*: 29%). A group summary of the clinical and behavioral characteristics of the patients is reported in Supporting Information Table S1.

### EEG Recording and Processing

A multichannel EEG cap was used to measure whole-scalp activity in each baseline recording. This consisted of resting-state measurement of 3 min under eyes open conditions, during which participants gazed at an empty black computer screen with their head comfortably positioned against at headrest. All EEG recordings were performed in standardized conditions during the patient’s clinical rehabilitation visits. Using a similar clinical EEG setup as in preceding work (Tokariev et al., 2018), scalp voltages were recorded with a 19 Ag/AgCl electrode cap (Electro-cap International, Inc., https://www.electro-cap.com) according to the 10–20 international system. The ground electrode was placed on the scalp, at a site equidistant between Fpz and Fz. Electrical signals were amplified with the Mitsar 21-channel EEG system (Mitsar-201, CE0537, Mitsar, Ltd. https://www.mitsar-medical.com), and all electrode impedances were kept under 5 kΩ. For online recording, electrodes were referenced to linked earlobes, and then the common average reference was calculated off-line before further analysis. EEG data was recorded at 250 Hz and then filtered with a 0.5–40 Hz band-pass filter off-line.

All EEG data were imported into the MATLAB toolbox EEGLAB v12 (https://sccn.ucsd.edu/eeglab/) for off-line processing. We used Infomax ICA decomposition to remove usual eye movement such as saccades or blinking (Jung et al., 2000). Recordings were further cleaned with an automated *z*-score-based method, using the FASTER plugin (Nolan, Whelan, & Reilly, 2010), rejecting 1-s epochs that deviated from the mean by more than 1.5 standard deviations, in light of frequent occurrences of involuntary head movements in the stroke patients. This strict selection procedure avoided an inflation of spurious increases in connectivity while preserving a large dataset in each individual. Finally, EEG discontinuities were minimized by utilizing Hann windowing (MATLAB hann() window function) on all clean epochs before concatenating them into continuous EEG.

### Source Space Measures of EEG Activity

Artifact-free EEG data were processed in MATLAB with the Brainstorm Toolbox (https://neuroimage.usc.edu/brainstorm/). In line with previous approaches using a similar EEG setup in clinical populations (Tokariev et al., 2018), we first computed a head model of the cortex surface for each EEG recording using the (symmetric) boundary element method from OpenMEEG (Gramfort, Papadopoulo, Olivi, & Clerc, 2010) and then estimated unconstrained cortical sources by using the minimum-norm sLORETA algorithm implemented in Brainstorm. To normalize sources across participants, we projected (warped) the sources from each participant onto the MNI/Colin27 template brain surface (Holmes et al., 1998). The 15,000 voxel source space was then divided into 68 cortical ROIs according to the Desikan–Killiany neuroanatomical atlas (Desikan et al., 2006). Temporal source activities across all the voxels in each ROI were then averaged and band-pass filtered in the following six frequency bands: delta 1–4 Hz, theta 4–8 Hz, alpha 8–12 Hz, beta 13–30 Hz, and gamma 30–45 Hz. For every subject, each frequency band was quantified in Brainstorm to examine differences in spectral power and FC between the patient and control groups.

### Spectral Power

Band-limited EEG power was estimated with a standard fast Fourier transform approach using Welch’s method (MATLAB pwelch() function) and a Hanning windowing function (4-s epoch, 50% overlap). Relative spectral power (i.e., % power) was calculated as the ratio of the mean power in a specific EEG band and the broadband power (1–45 Hz). Multiple comparison correction was performed using the FDR option in the Brainstorm Toolbox.

### Functional Connectivity

A single time course was constructed for each ROI, which was then defined as a *node* in a network graph. Connectivity between nodes was subsequently estimated using the amplitude envelope correlation, yielding a 68 × 68 node adjacency matrix. The first step in estimating the amplitude envelope correlation is to compensate for spatial leakage confounds by using a bidirectional orthogonalization procedure (Hipp, Hawellek, Corbetta, Siegel, & Engel, 2012) to remove all shared signal at zero lag between filtered EEG signals. After this, we computed the instantaneous amplitude (i.e., envelope) across time for each frequency band by using the absolute value of the Hilbert transform. Finally, the linear correlation between the amplitude time series of each node pair was calculated using the Pearson correlation coefficient (Brookes et al., 2011). For visualization of group differences (i.e., Figure 1), we reported the absolute difference in Pearson correlation (*r*) which may be regarded as a standardized measure of effect size (Nakagawa & Cuthill, 2007).

### Statistical Analyses

Source space (voxel-wise) statistical comparisons of band-limited spectral power were performed using the Brainstorm Toolbox via independent two-tailed *t* tests with a *p* < 0.05 threshold. Separately, we used the GraphVar Toolbox (Kruschwitz, List, Waller, Rubinov, & Walter, 2015) for statistical comparisons of network connectivity using intergroup *t* tests with a *p* < 0.05 threshold. Individual neglect severity was measured by performance on the cancellation test and subsequently used in a between-subject regression analysis (with a *p* < 0.05 threshold).

Our statistical analyses followed a stepwise approach. First, we performed a direct comparison of whole-brain connectivity changes in stroke patients versus healthy controls, regardless of neglect severity. This allowed us to optimize our statistical power by considering the whole sample data and avoid a dichotomous categorization of neglect presence versus absence based on arbitrary criteria, given that neglect diagnosis may vary according to the test used (Checketts et al., 2020; Verdon et al., 2010) and that many of our patients showed some neglect deficits in one task but not in others. Next, we performed a regression analysis to identify those connections whose strength varied as a function of the severity of neglect deficits. The specificity of this relationship was further ensured by a final conjunction test (Nichols, Brett, Andersson, Wager, & Poline, 2005) to isolate neglect-related changes among the stroke-related anomalies in connectivity patterns. Impaired visuospatial attention was quantified by the absolute number of omitted targets (i.e., errors) within the left and right hemifields during the cancellation test in each individual patient. Please note we preferred the absolute rather than the relative number of omissions (e.g., difference between left and right) as the latter did not reliably reflect the stroke severity and extent of network damage in EEG. This is because stroke-related lateralized damage in brain network should induce a proportional degree of lateralized behavioral deficit, while a numerically similar difference in relative number (left minus right) could occur with different absolute values and different extent of brain damage. A similar quantitative analysis of brain–behavior relationships using deviation severity on the line bisection test did not yield any significant results and was not further reported.

For all tests, and to statistically correct for multiple comparisons in FC measures, we used the NBS (Zalesky, Fornito, & Bullmore, 2010) based on MATLAB code from the Brain Connectivity Toolbox (brain-connectivity-toolbox.net). We performed *n* = 5,000 permutations to generate a null network model based on a random shuffle of all connections. Basically, the NBS is used to control the family-wise error rate when the null hypothesis is tested independently at each of the N(N-1)/2 edges comprising the connectivity matrix. The NBS may provide greater statistical power than conventional procedures such as FDR, when the set of edges at which the null hypothesis is rejected constitutes large component(s). The theoretical basis of the NBS is to consider the pairwise similarity matrix of functional connections by using the framework of graph theory, insofar as conventional cluster statistics are applied to a graph structure, with the main difference that graph components (i.e., significantly interconnected nodes) play the role of voxel clusters. This proceeds as follows. First, the test statistic computed for each link is thresholded to construct a set of suprathreshold links. Any connected structures, or components in graph terminology, that may be present in the set of suprathreshold links are then identified. Lastly, using permutation tests, a *p* value is assigned to each identified component by indexing its size with the null distribution of maximal component size. As a result, in the NBS framework, it is not possible to declare individual links (pairwise connections) as being significant, but only the network component to which they belong can be declared significant. Hence, the individual (i.e., link level) connection *t* values were reported mainly indicatively, together with *d* as the absolute difference in Pearson correlation (*r*), which may be regarded as a standardized measure of effect size.

### Data Availability

Anonymized EEG data and MATLAB analysis scripts that were used in this study are available from the corresponding author upon request.

## ACKNOWLEDGMENTS

The authors would like to thank all the participants and their families, the neuropsychologists, all members of the neuropsychology unit, and the general rehabilitation team of the Clinique Romande de Réadaptation and Vallée de Coeur Institute in Sion for their help and useful advice.

## SUPPORTING INFORMATION

Supporting information for this article is available at https://doi.org/10.1162/netn_a_00210.

## AUTHOR CONTRIBUTIONS

Tomas Ros: Conceptualization; Formal analysis; Methodology; Visualization; Writing – original draft; Writing – review & editing. Abele Michela: Data curation; Project administration. Anaïs Mayer: Data curation; Methodology. Anne Bellmann: Conceptualization; Investigation; Project administration. Philippe Vuadens: Conceptualization; Data curation; Investigation; Project administration. Victorine Zermatten: Investigation; Project administration. Arnaud Saj: Conceptualization; Funding acquisition; Investigation; Project administration; Resources. Patrik Vuilleumier: Conceptualization; Funding acquisition; Supervision; Writing – review & editing.

## FUNDING INFORMATION

This study was generously supported by grants from the Leenaards Foundation, as well as the EU Marie-Curie COFUND program BRIDGE (Grant no. 267,171), Geneva University Hospitals (HUG), Société Académique de Genève, and the Swiss National Science Foundation (SNF; Grant no. 320030-166704).

## TECHNICAL TERMS

Electroencephalogram (EEG): A recording of brain activity where non-invasive sensors are attached to the scalp to detect the electrical signals produced by neurons.
Functional network connectivity (FNC): A general term for the degree of functional coupling between brain regions, here measured by the amplitude envelope correlation (AEC).
Network-based statistic (NBS): A statistical-correction method for comparing network connectivity matrices based on the largest connected components.
Hyperconnectivity: An increase in the amplitude envelope correlation (AEC) between brain regions.
Hypoconnectivity: A decrease in the amplitude envelope correlation (AEC) between brain regions.
Visuospatial neglect: Cognitive deficit characterized by reduction or loss of spatial awareness for the contralesional space, most commonly after unilateral stroke or brain injury.
Low-resolution electromagnetic tomography analysis (sLORETA): A method of mathematically analyzing multiple EEG signals from across the scalp to determine their anatomical source within the brain.
Amplitude envelope correlation (AEC): The correlation coefficient between the instantaneous amplitude fluctuations of two brain regions.
