# Supplementary material for: Disruption of large-scale electrophysiological networks in stroke patients with visuospatial neglect
Source: Netw Neurosci. 2022 Feb 1;6(1):69–89. doi: 10.1162/netn_a_00210 (PMC8959119; doi:10.1162/netn_a_00210)
Supplement: Supplementary file 1 [file netn-06-69-s001.pdf]

Ros, T., Michela, A., Mayer, A., Bellmann, A., Vuadens, P., Zermatten, V., Sai, A. & Vuilleumier, P. (2022). Supporting information for "Disruption of large-scale electrophysiological networks in stroke patients with visuospatial neglect." *Network Neuroscience, 6*(1), 69–89. https://doi.org/10.1162/netn_a_00210

Supplementary Material

	Mean	Standard deviation	Range
Age (years)	59.4	11.3	32 - 79
Sex (M/F)	13 M / 2 F	-	-
Time since Stroke Onset (months)	7.5	5.3	1 - 24.5
Hemianopia (%)	27% (4/15)	-	-
Left omission errors (%)	77%	29%	13% - 100%,
Right omission errors (%)	23%	23%	0%– 67%
Central omission errors (%)	54%	42%	0% - 100%
Total omission errors (%)	51%	26%	6% – 86%

Suppplementary Table 1: **Summary data of the stroke patient sample (n=15).** The table shows the group mean, standard deviation and range (i.e. minimum and maximum values) for clinical and behavioral variables.

Ros, T., Michela, A., Mayer, A., Bellmann, A., Vuadens, P., Zermatten, V., Sai, A. & Vuilleumier, P. (2021). Supporting information for "Disruption of large-scale electrophysiological networks in stroke patients with visuospatial neglect." *Network Neuroscience*. Advance publication. https://doi.org/10.1162/netn_a_00210

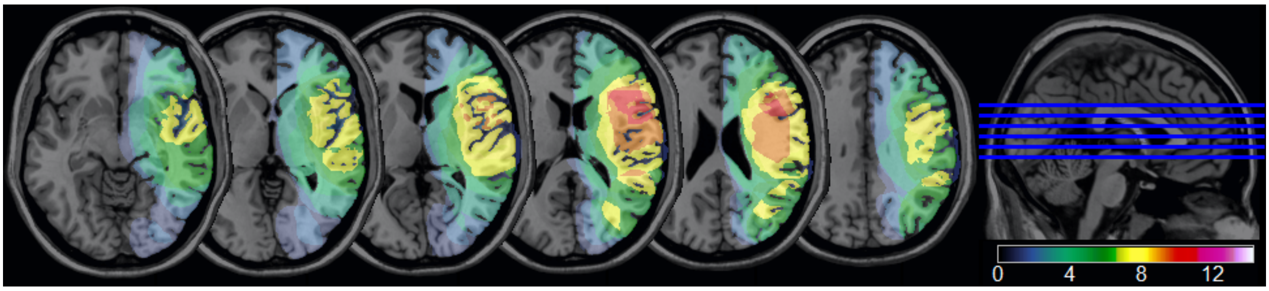

Supplementary Fig. S1: **Average structural MRI lesion maps of the 15 stroke patients** with hemineglect. Colors code for the number of patients with damage to a particular voxel location. Lesions were widely distributed in the right hemisphere, with the strongest overlap in fronto-insular regions (red colour, peak coordinates [x= 42, y= -2, z= 14]), affected in a maximum of 10 patients.

Ros, T., Michela, A., Mayer, A., Bellmann, A., Vuadens, P., Zermatten, V., Sai, A. & Vuilleumier, P. (2021). Supporting information for "Disruption of large-scale electrophysiological networks in stroke patients with visuospatial neglect." *Network Neuroscience*. Advance publication. https://doi.org/10.1162/netn_a_00210

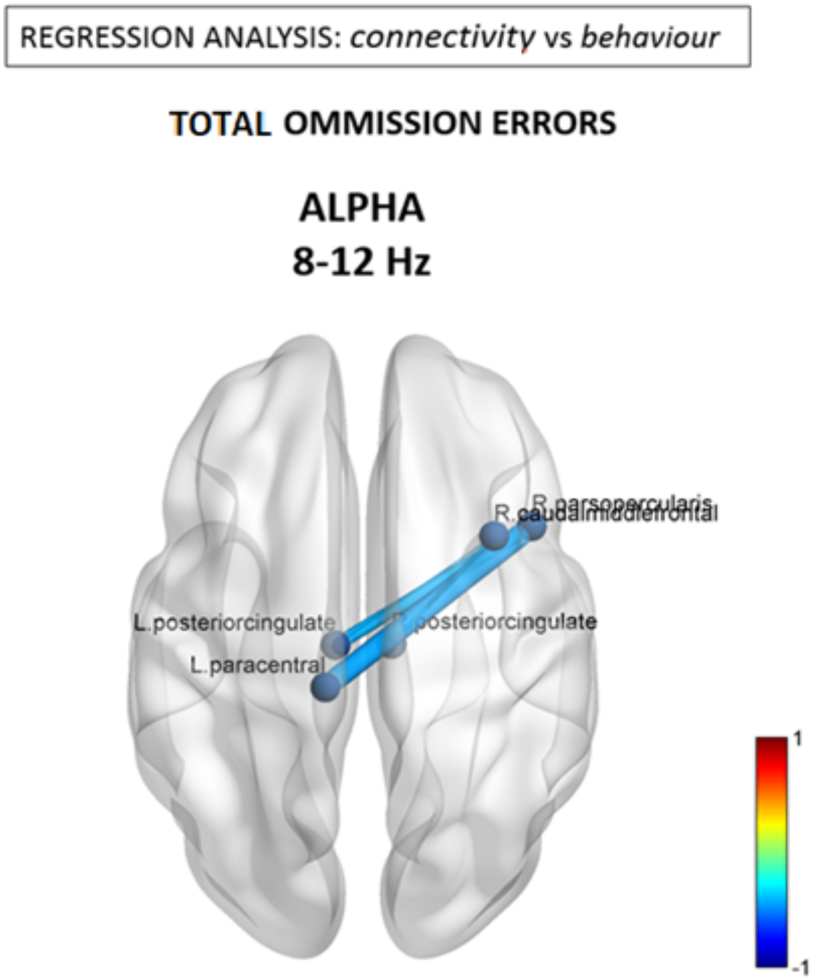

Supplementary Fig. S2: **Alpha (8-12 Hz) connectivity as a function of neglect severity.** Depicted connections correspond to changes in individual pairwise FNC correlating with the *total* number of left and right omissions during the cancellation task. Red/blue values indicate statistically significant beta coefficients ($p < 0.05$ Network Based Statistic (NBS) corrected)

Ros, T., Michela, A., Mayer, A., Bellmann, A., Vuadens, P., Zermatten, V., Sai, A. & Vuilleumier, P. (2021). Supporting information for "Disruption of large-scale electrophysiological networks in stroke patients with visuospatial neglect." *Network Neuroscience*. Advance publication. https://doi.org/10.1162/netn_a_00210

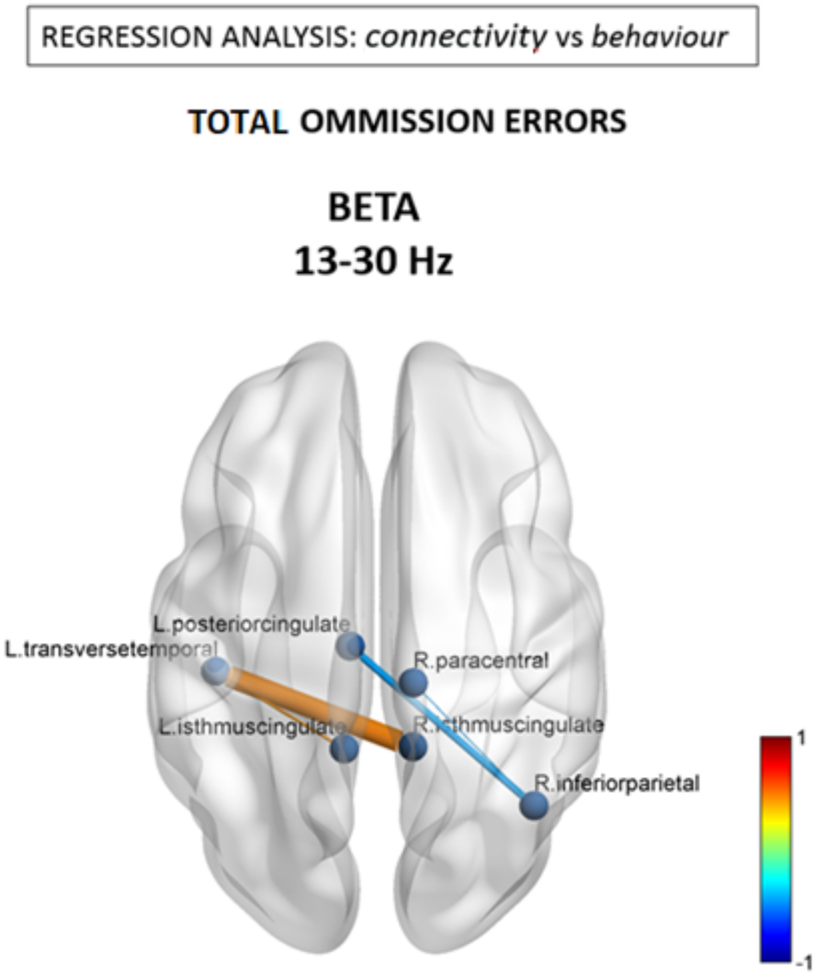

Supplementary Fig. S3: **Beta (13-30 Hz) connectivity as a function of neglect severity.** Depicted connections correspond to changes in individual pairwise FNC correlating with the *total* number of left and right omissions during the cancellation task. Red/blue values indicate statistically significant beta coefficients ($p < 0.05$ Network Based Statistic (NBS) corrected)

Ros, T., Michela, A., Mayer, A., Bellmann, A., Vuadens, P., Zermatten, V., Sai, A. & Vuilleumier, P. (2021). Supporting information for “Disruption of large-scale electrophysiological networks in stroke patients with visuospatial neglect.” *Network Neuroscience*. Advance publication. https://doi.org/10.1162/netn_a_00210

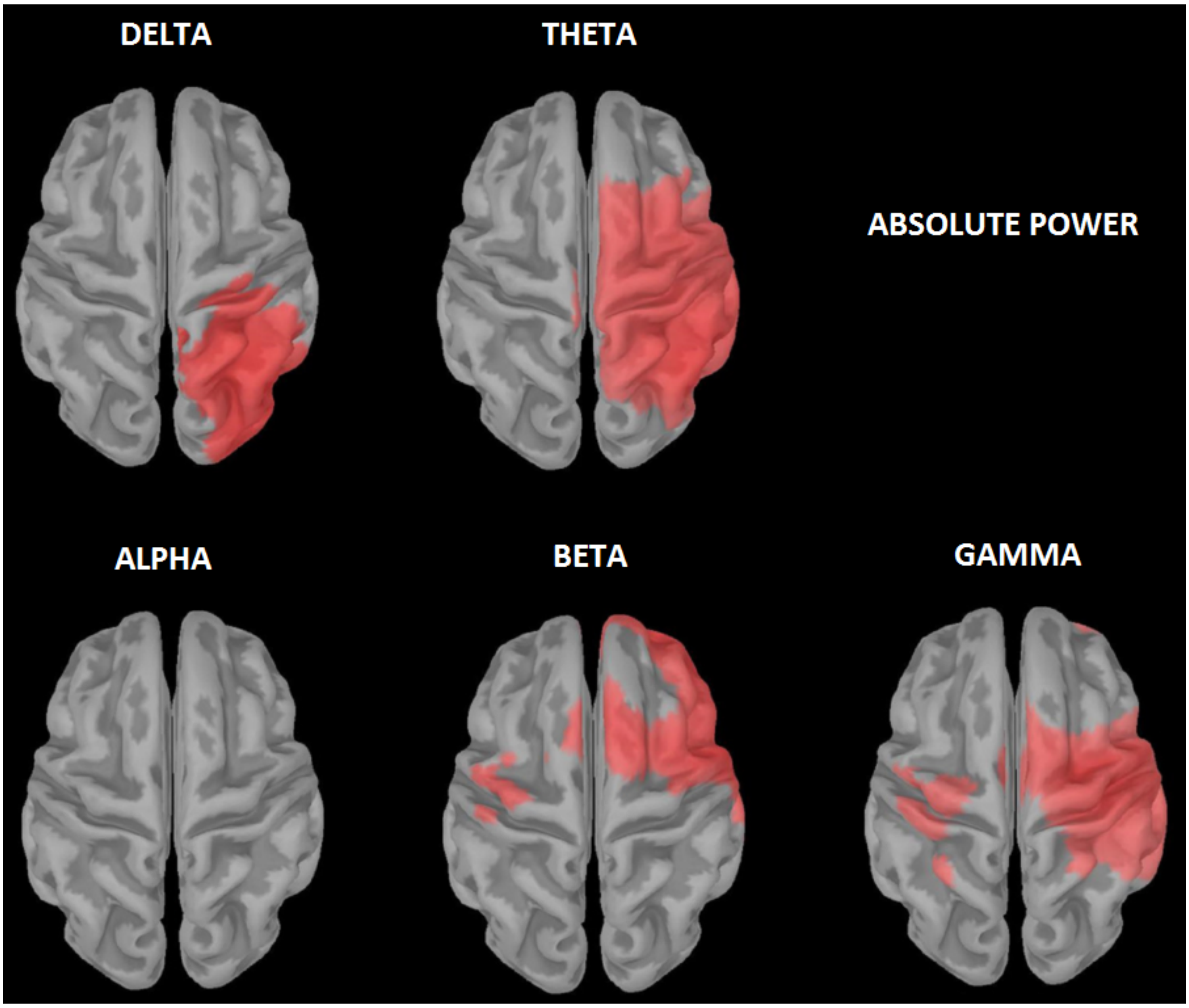

Supplementary Fig. S4. **Absolute spectral power.** T-values of statistical differences in sLORETA relative source-power between hemineglect patients and controls. *Red colour* indicates greater power for patients, while *blue colour* indicates greater power for controls ($p < 0.05$ FDR corrected).

Ros, T., Michela, A., Mayer, A., Bellmann, A., Vuadens, P., Zermatten, V., Sai, A. & Vuilleumier, P. (2021). Supporting information for "Disruption of large-scale electrophysiological networks in stroke patients with visuospatial neglect." *Network Neuroscience*. Advance publication. https://doi.org/10.1162/netn_a_00210

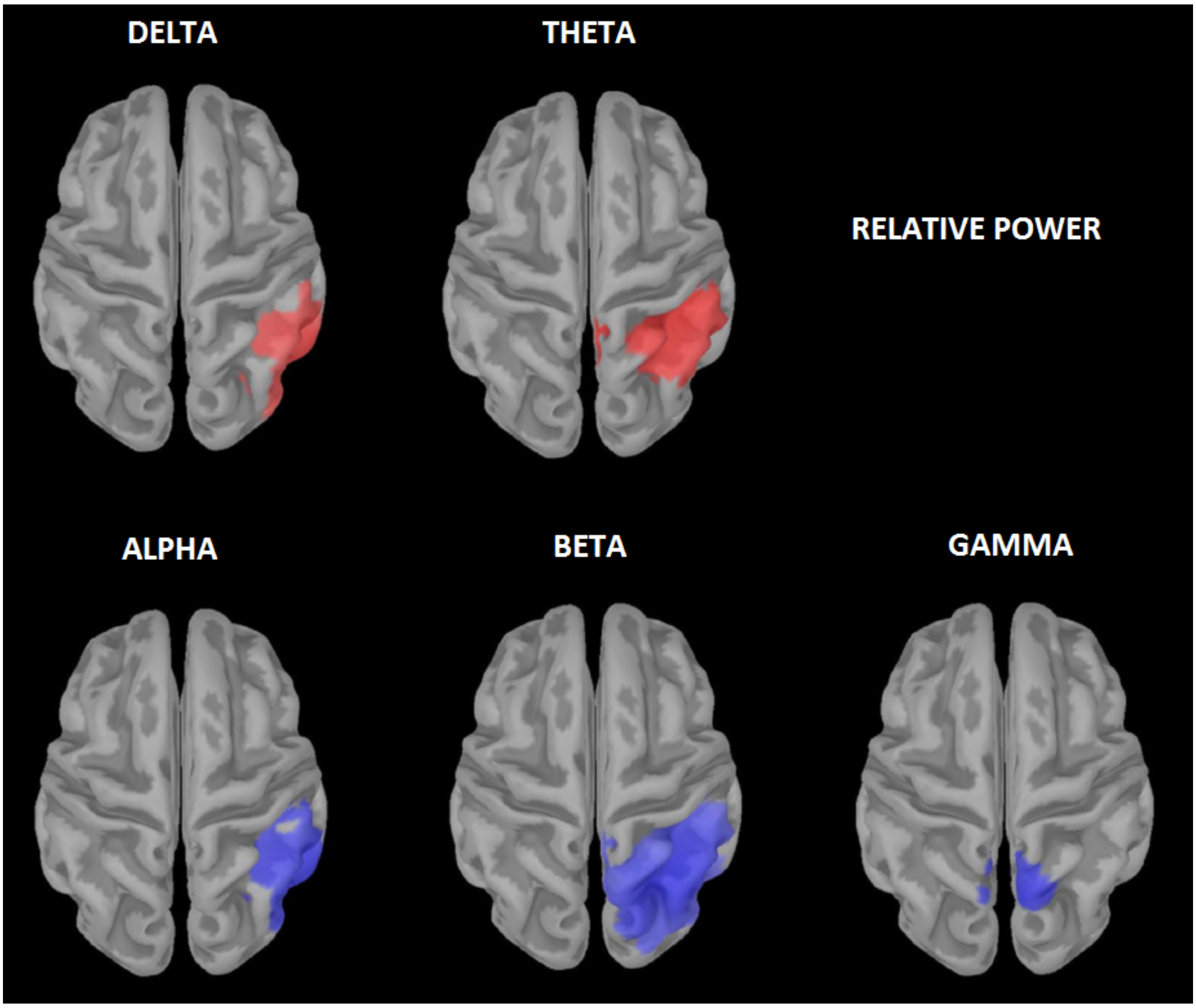

Supplementary Fig. S5. **Relative spectral power.** T-values of statistical differences in sLORETA relative source-power between hemineglect patients and controls. *Red colour* indicates greater power for patients, while *blue colour* indicates greater power for controls (p < 0.05 FDR corrected).

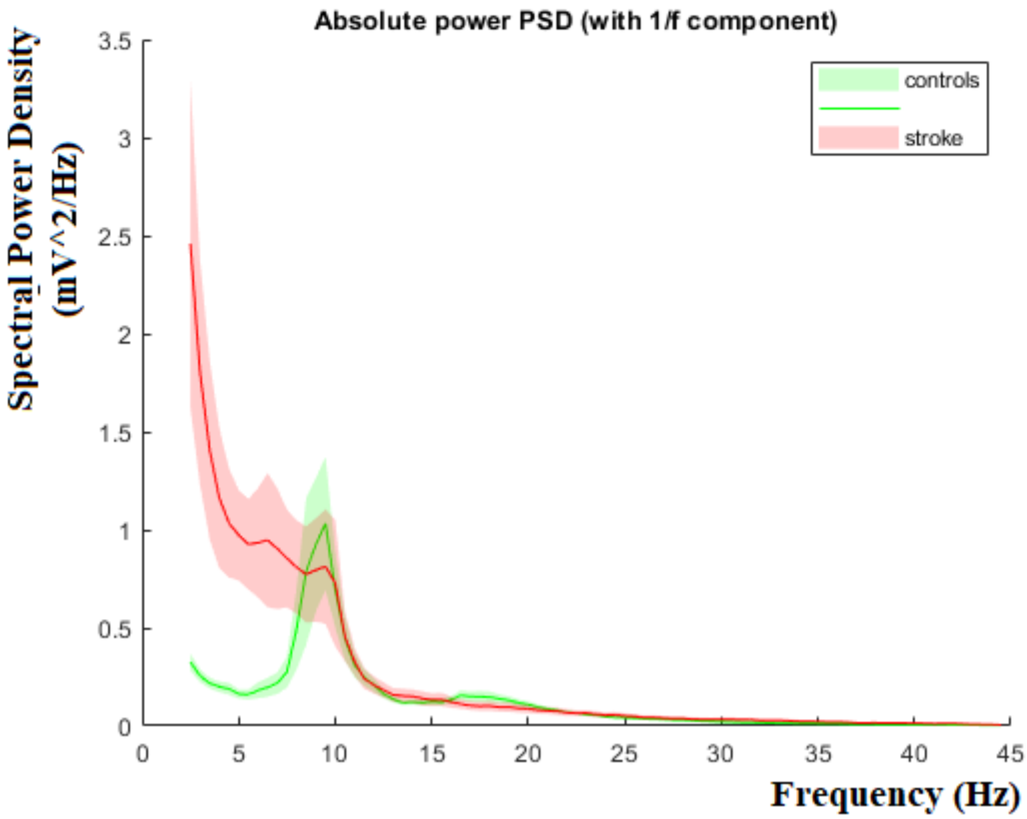

***Supplementary Fig. S6*. Absolute Spectral Power Density** (PSD) plot of EEG power vs frequency between hemineglect patients and controls, at right posterior parietal cortex (current-source density montage, electrode P4). *Red colour* indicates absolute power for patients, while *green colour* indicates absolute power for controls.

Ros, T., Michela, A., Mayer, A., Bellmann, A., Vuadens, P., Zermatten, V., Sai, A. & Vuilleumier, P. (2021). Supporting information for "Disruption of large-scale electrophysiological networks in stroke patients with visuospatial neglect." *Network Neuroscience*. Advance publication. https://doi.org/10.1162/netn_a_00210

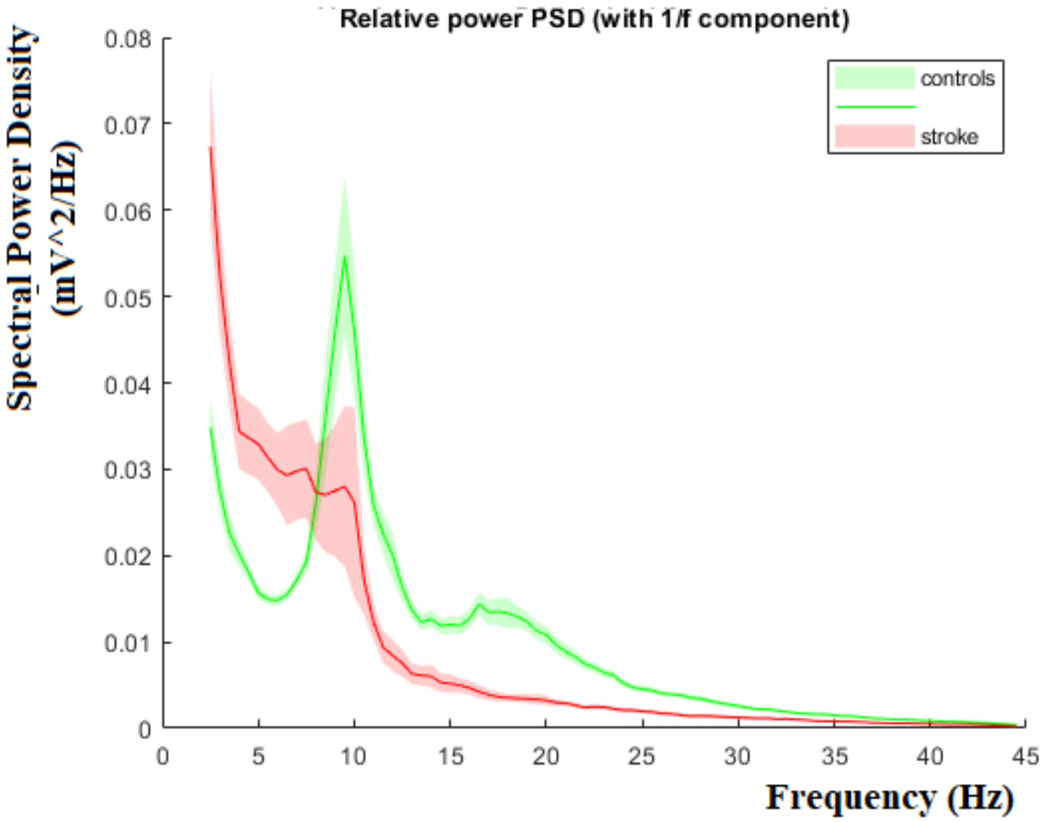

Supplementary Fig. S7. **Relative Spectral Power Density** (PSD) plot of EEG power vs frequency between hemineglect patients and controls, at right posterior parietal cortex (current-source density montage, electrode P4). *Red colour* indicates relative power for patients, while *green colour* indicates relative power for controls.

Ros, T., Michela, A., Mayer, A., Bellmann, A., Vuadens, P., Zermatten, V., Sai, A. & Vuilleumier, P. (2021). Supporting information for "Disruption of large-scale electrophysiological networks in stroke patients with visuospatial neglect." *Network Neuroscience*. Advance publication. https://doi.org/10.1162/netn_a_00210

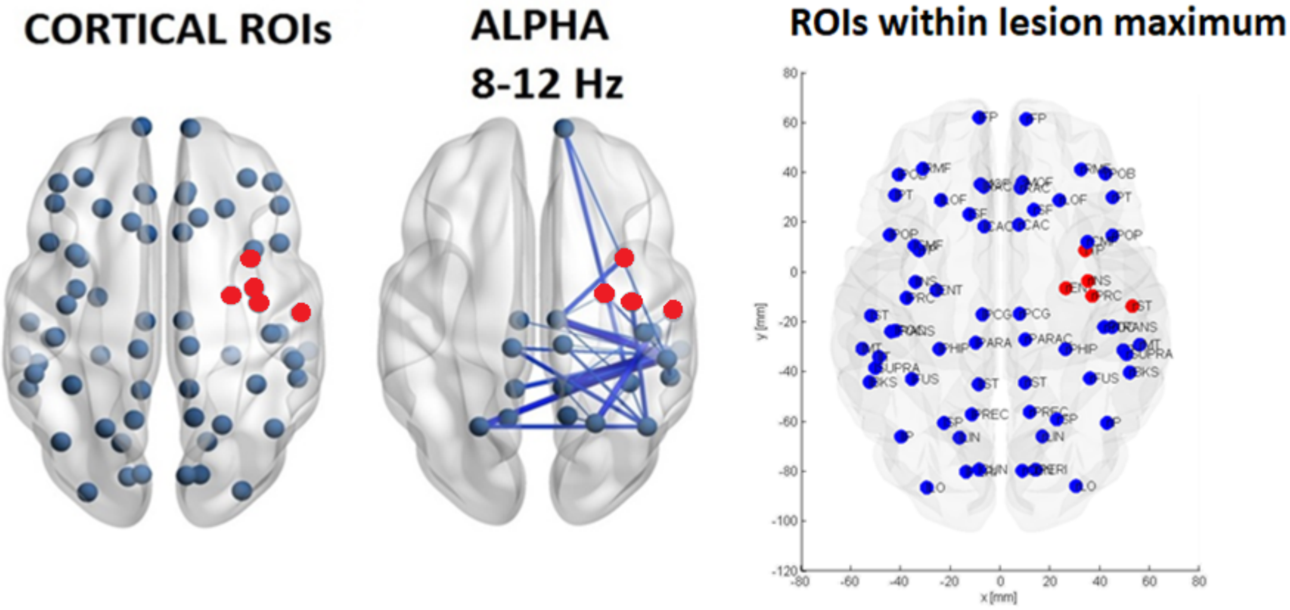

Supplementary Fig. S8: **Network nodes within locus of maximum lesion** with peak coordinates [x= 42, y= -2, z= 14]). Affected nodes are depicted in *red colour* in the 3rd panel with MNI coordinates; 1st and 2nd panels are from Fig. 1 for comparison. See also Supplementary Figure 3 for structural MRI image of lesions.
